# Development of a Lensless Compact Optical System for Ultrasensitive Nanomaterial Detection in Liquid Media via Exploiting Multiple Light Scattering

**DOI:** 10.1002/nap2.70193

**Published:** 2026-07-05

**Authors:** Jeonggyo Kim, Youngsoo Kim, Jun‐Won Kook, Hae‐Nam Choi, Kyoungman Cho, In‐Hwan Lee, Jongkook Hwang, Sungju Yu, Chung‐Yul Yoo, Jonghee Yoon

**Affiliations:** ^1^ Department of Energy Systems Research Ajou University Suwon South Korea; ^2^ Ajou Energy Science Research Center Ajou University Suwon South Korea; ^3^ The Wave Talk. Inc. Daejeon South Korea; ^4^ Department of Chemistry Ajou University Suwon South Korea; ^5^ Department of Chemical Engineering Ajou University Suwon South Korea; ^6^ Department of Physics Ajou University Suwon South Korea

**Keywords:** laser speckle imaging, multiple light scattering, nanoparticle, sensor

## Abstract

Accurate detection of nanoparticles in liquids is essential across drug delivery, diagnostics, and environmental monitoring. Dynamic light scattering (DLS), the current gold‐standard technique, relies on single‐scattering events and requires prior knowledge of sample temperature and optical properties, limiting its sensitivity at low particle concentrations and increasing system cost and complexity. Here, we present a lensless, cost‐effective speckle‐based method that exploits multiple light scattering to overcome these limitations. A custom‐machined aluminum sample holder uniformly illuminates the entire sample volume with a randomized coherent light field via repeated diffuse reflections. Subtle nanoparticle motions modulate the phase of the scattered light, producing time‐varying speckle patterns captured by a CMOS camera without any imaging optics. Temporal correlation analysis of the speckle time series quantifies nanoparticle dynamics without requiring prior sample information. The method detects nanoparticles at concentrations as low as 10^5^ particles/mL, which is at least 10^3^–10^4^‐fold below the DLS detection limit for sub‐200 nm particles. Under fixed‐concentration conditions, an empirical calibration model achieves semi‐quantitative size determination in the 100–200 nm range with 95% prediction intervals within ± 7 nm. The proposed platform therefore offers a versatile, field‐deployable solution for ultrasensitive nanoparticle characterization in liquid media.

## Graphical abstract

This work presents a lensless speckle‐based platform that exploits multiple light scattering for precise nanoparticle detection in liquid media. The proposed method enables ultrasensitive detection at concentrations far below conventional DLS limits and provides semiquantitative size estimation, offering a cost‐effective and field‐deployable approach for advanced nanoparticle analysis.

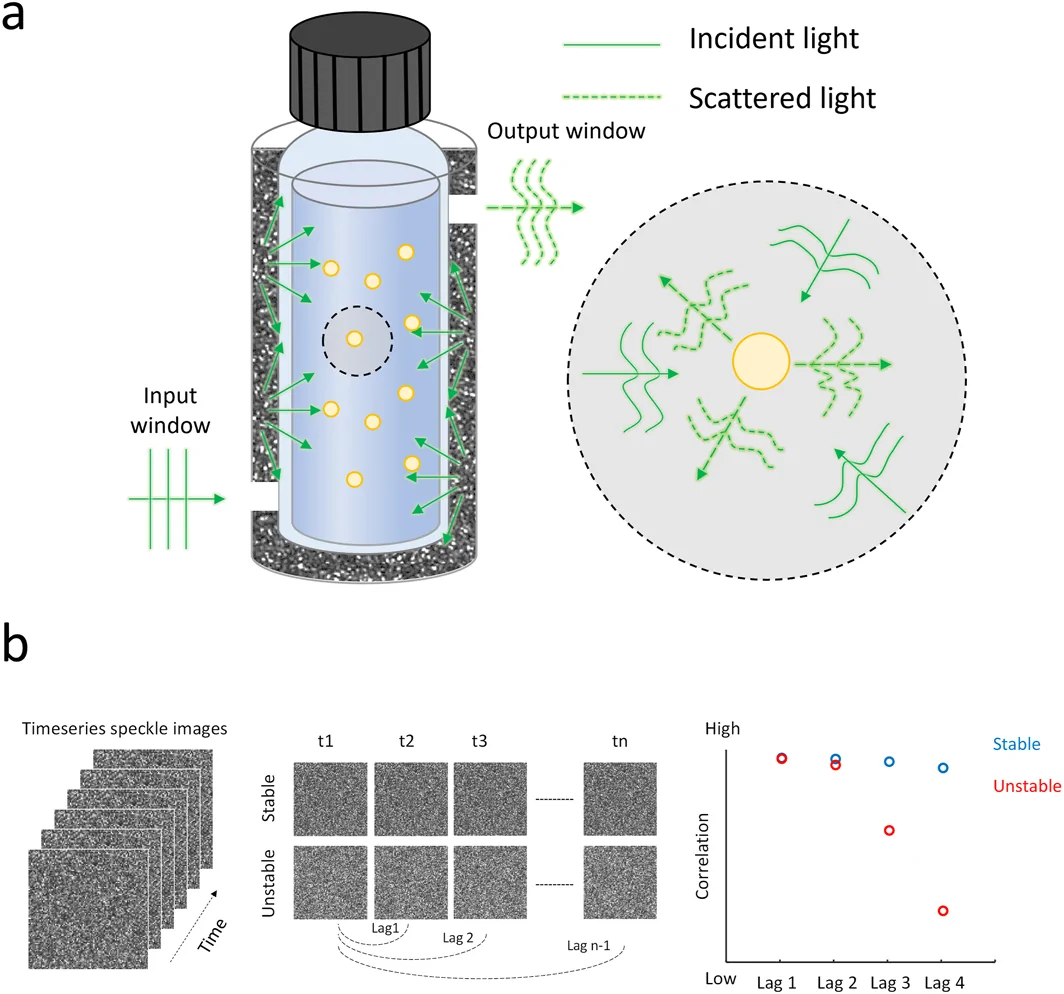

## 1 Introduction

Nanomaterials dispersed in liquids play central roles in various applications, including drug delivery systems [1, 2], mRNA vaccines [3, 4], food science [5], diagnostic assays [6, 7], and water quality monitoring [8, 9]. As the demand for nanoparticle‐related technologies grows, accurate characterization of nanoparticles in liquid media becomes essential for guaranteeing stable nanoparticle synthesis, safety, and functional performance.

Dynamic light scattering (DLS) has been widely used as the gold‐standard technique for nanoparticle analysis in both academic research [10, 11, 12] and industries [13, 14, 15]. Despite its broad applicability in nanoparticle analysis, DLS fundamentally relies on single‐scattering events and assumes dilute, homogeneous particle suspensions [16, 17]. Consequently, it faces critical limitations such as sensitivity breakdown at low nanoparticle concentrations due to insufficient scattered light intensity and inaccuracy in high‐concentration samples due to multiple light scattering [18]. Moreover, DLS requires a photodetector for the fast measurement of light intensity variations caused by Brownian motion of particles. Thus, the photodetector is usually placed at a specific angle [19] considering a single scattering event of a sample and the measured light signals were analyzed based on Stokes–Einstein equation requiring information about temperature and diffusion coefficient [17], which cause issues in the analysis accuracy of unknown samples without prior information. Consequently, conventional DLS instruments are bulky and expensive, requiring dedicated analysis software that further increases the overall system cost. To overcome multiple light scattering and angle‐dependency issues, diffusing‐wave spectroscopy has been developed [20, 21]. It allows quantitative nanoparticle analysis under multiple‐scattering conditions without angle dependency; however, extracting particle size from DWS data requires fitting the measured correlation function to an analytical transport model that assumes a known sample geometry, mean free path, and particle scattering coefficient. These parameters are difficult to determine independently for unknown samples, making DWS impractical for routine nanoparticle characterization without extensive prior characterization of the sample. Several advanced techniques have been developed to push nanoparticle detection sensitivity beyond DLS. iSCAT achieves single‐particle sensitivity via interferometric detection but requires precision optics and surface‐immobilized samples, limiting its use for bulk liquid‐phase sensing [22]. MDSS improves DLS analysis robustness by extracting particle size from speckle time series without explicit optical model parameters [23], yet remains subject to similar concentration detection limits as conventional DLS. Image‐based DLS methods have also been proposed in which a 2D image sensor replaces the photomultiplier tube of conventional DLS and particle size is extracted from the temporal correlation of consecutive scattering images [24]; however, these approaches operate within the single‐scattering framework and are subject to concentration detection limits comparable to those of conventional DLS. However, there are still unmet needs for alternative approaches with improved sensitivity, lower cost, and more robust measurement of nanoparticle characteristics without prior information such as temperature and sample's optical properties.

In this study, we employed laser speckle imaging to address two practical limitations of conventional approaches: the need to measure light signals at a specific angle corresponding to single‐scattering event by samples, and requirement information such as temperature and detailed optical properties of nanomaterials for quantitative analysis. Laser speckles arise from constructive and destructive interference of light, and it is known that when highly coherent light such as a laser undergoes multiple scattering events, speckle grain sizes become comparable to the diffraction limit of the optical system [25, 26, 27]. Although laser speckle images seem like complicated noise, they are the outcome of a deterministic process governed by the interaction between light and the medium; therefore, when the medium and the light source remain stable over time, a consistent speckle pattern is produced. Exploiting these properties, wavefront‐shaping methods have demonstrated that shaping the incident wavefront enables the formation of a well‐defined focus inside a scattering medium, which could overcome scattering issues in turbid media [28, 29]. Analyzing temporal dynamics of speckle patterns allows the investigation of subtle changes in the medium, which has been applied to vascular imaging, blood‐flow velocimetry, and microorganism detection. Thus, laser speckle images encode detailed information about the medium and are emerging as a powerful platform for sensing technologies.

We developed a laser‐speckle–based optical technique for enhancing the sensitivity of detecting nanomaterials dispersed in liquids by deliberately maximizing multiple light scattering effects. We propose and demonstrate a method that exploits multiple light scattering for enhanced nanomaterial detection sensitivity without prior information of imaging environments and sample optical properties. We made a metal‐coated sample holder to induce multiple‐light scattering of incident light, producing time‐varying laser speckle images captured by a CMOS camera placed at an arbitrary position. Temporal correlation analysis of speckle images enhances nanomaterial detection sensitivity by capturing the subtle intensity fluctuations induced by nanoparticle motion encoded within the speckle dynamics. The proposed method enables the discrimination of low concentrations of nanoparticles, which cannot be achievable via the commercial DLS equipment. Because the optical system does not require any optical lens to capture laser speckle images—relying on a laser source, a sample holder which could induce multiple light scattering, and a CMOS camera—the proposed optical system is both cost‐effective and simple. Thus, the proposed method would be a versatile tool when the simple and sensitive analysis of nanomaterials in liquid is required without prior information about a sample.

## 2 Results and Discussion

Figure 1a shows a schematic illustration of the key idea of the proposed method. A metal‐coated sample holder that induces strong multiple light scattering at its surface is used so that the incident laser light undergoes multiple scattering events which allows to fill the entire sample area with incident light. As a result, light with random phases illuminates the nanoparticles dispersed in the liquid from various directions, and the light scattered by the nanoparticles propagates in all directions. Even small motions of the nanoparticles then lead to changes in the phase of the scattered light, which in turn modifies constructive and destructive interference of the light reaching the camera. Consequently, the recorded speckle patterns encode information about the nanoparticles.

**FIGURE 1:**
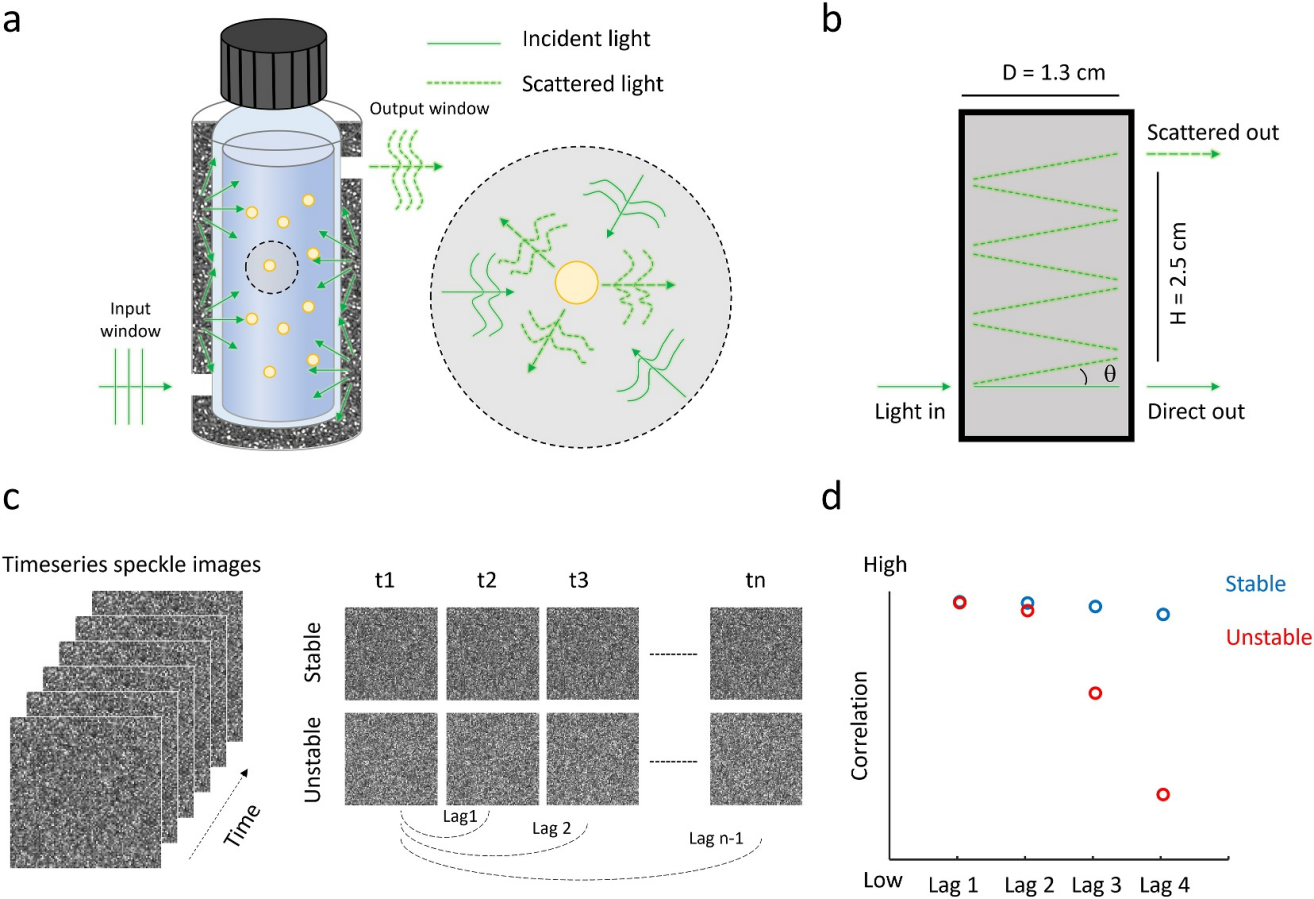
Schematics of multiple light scattering‐based nanomaterial sensing method. (a) Concept of harnessing multiple‐light scattering for the ultrasensitive detection of nanomaterials in liquid. The input and output windows are located on opposing side walls of the aluminum holder; the lensless CMOS camera is placed laterally facing the side output aperture (not the top of the vial). Each nanomaterial is illuminated by surrounding light field generated via multiple‐light scattering, then it produces scattered light fields in all directions, resulting in changes in interference patterns at the detector. (b) Theoretical analysis of optical‐path‐length enhancements obtained by considering only the law of reflection at metallic surfaces. (c) Image‐processing pipeline for calculating temporal correlation of timeseries speckle images at different time lags. (d) Conceptual correlation analysis of speckle images obtained at stable (blue) and unstable (red) nanomaterial conditions.

Supporting Information S1: Figure S1a shows the optical setup for laser speckle imaging, consisting of a 532 nm laser, sample holder, and CMOS camera. The CMOS camera was mounted without lenses or imaging optics, directly facing the output aperture of the sample holder. Speckle patterns formed on the sensor plane via free‐space interference of the multiply‐scattered coherent light field, with speckle grain size increasing proportionally with camera‐to‐sample distance in accordance with far‐field diffraction (Supporting Information S1: Figure S1b). As speckle images were the outcome of optical field interferences scattered from the sample holder, sample vial, and sample, it is not affected by any distortion caused by the curvature of the sample vial.

To verify that the multiple light scattering induced by the sample holder can effectively cover the entire sample volume, we performed a simple calculation. To estimate the lower bound of the optical path length (OPL) enhancement, we modeled the sample holder as a two‐dimensional rectangular cross‐section and considered only specular reflection (i.e., the law of reflection) at the cavity walls, deliberately excluding diffuse scattering. Then, we estimated how much the OPL is extended before the light reaches the camera. For the realistic estimation of OPL enhancement, the actual dimensions of the sample holder (13 × 13 × 25 mm) were used.

When a laser was illuminated perpendicularly through the input port and the beam size was sufficiently larger than the port size, the light passing through the port spreads owing to Fraunhofer diffraction.

(1) $$\theta \approx \frac{\lambda }{d}$$

Equation (1) is the well‐known formula for Fraunhofer diffraction, where *θ* is the diffraction angle, *λ* is the wavelength of the laser, and *d* is the diameter of the input port. The wavelength of the laser used was 532 nm, and the input port had a diameter of 0.35 cm. Hence, the Fraunhofer diffraction angle was as small as 0.009°. However, this implies that the light reaching the actual exit port must travel a very long distance. Therefore, if we define the OPL of light that goes straight from the incident hole by its diameter and is emitted directly as OPL_0, and the effective OPL of light that travels a height *H* before exiting through the emission port as OPL_eff. Then, the height traversed at once by light spread out by Fraunhoffer diffraction can be expressed as follows:

(2) $$h=\text{OP}L_{0}\;\tan\;\theta$$

where the optical path traveled by the light to the other side is represented as follows:

(3) $$L=\frac{\text{OP}L_{0}}{\cos\;\theta }$$

According to these two equations, the total optical path traveled by the light as it moves vertically by *h* several times to reach the outlet height *H* from the inlet to the outlet can be expressed as follows:

(4) $$\text{OP}L_{\text{eff}}=L\cdot \frac{H}{h}$$

(5) $$\text{OP}L_{\text{eff}}=\frac{H}{\sin\;\theta }$$

When the actual parameters are substituted and calculated (Fraunhofer diffraction angle = 0.009°and *H* = 2.5 cm ), the distance light travels on the plane until it reaches the outlet is 15,915 cm, which is 39,789 times greater than the actual diameter of 1.3 cm. In a realistic three‐dimensional cylindrical geometry, with multiple scattering from both the holder and the sample, the light experiences a much longer effective path length before reaching the camera. Thus, the measured speckle image can be regarded as containing information integrated over the entire sample volume.

To provide a theoretical foundation for the sensitivity enhancement conferred by the custom sample holder, we performed Monte Carlo and Finite‐difference time‐domain (FDTD) simulations characterizing the light field distribution inside the cavity.

Monte Carlo simulations modeled the spatial distribution of photon paths within the cylindrical aluminum cavity under two limiting surface conditions. When the inner surface acts as a specular reflector, the incident laser beam (incident angle 0.1°) propagates along a narrow geometric path, and the illuminated volume remains confined to a small fraction of the total cavity cross‐section (Supporting Information S1: Figure S2a). When the surface instead behaves as a diffuse reflector, photons undergo repeated random reflections and redistribute throughout the entire cavity volume (Supporting Information S1: Figure S2b). This transition from specular to diffuse multiple scattering dramatically increases the effective optical path length experienced by each photon, which is the direct physical origin of the sensitivity enhancement: nanoparticles located anywhere within the cavity volume perturb the multiply‐scattered light field, and every such perturbation contributes to the temporally fluctuating speckle pattern recorded by the camera.

FDTD simulations independently confirmed this picture at the electromagnetic level. When a dipole pulse is introduced at one end of the cavity, the resulting electric field propagates and uniformly fills the entire cavity cross‐section within a short propagation distance (Supporting Information S1: Figure S3), consistent with the spatially uniform photon distribution predicted by the Monte Carlo model. The agreement between the two simulation approaches validates the physical interpretation that the diffuse‐scattering cavity creates a spatially homogeneous light field throughout the sample volume.

This spatially uniform illumination has two direct consequences for nanoparticle detection sensitivity. First, every nanoparticle in the sample volume contributes to the speckle signal regardless of its position, unlike in DLS where only particles within the focused scattering volume are detected. Second, the extended effective path length increases the phase shift accumulated per nanoparticle displacement, amplifying the temporal decorrelation signal for a given particle motion.

As time‐varying speckle images are directly related to the nanomaterials within the liquid, it was analyzed via temporal correlation calculation which shows how speckle patterns are stable over time. Figure 1c shows schematic temporal correlation analysis process to quantify time‐varying speckle images. By computing the correlation as a function of time lag, nanomaterials with different conditions (stable vs unstable) could be discriminated via correlation values. Nanomaterial conditions refer to the extent to how nanomaterials alter scattering properties of the medium. Thus, for nanomaterials of the same size, lower concentrations correspond to a more stable condition, whereas for a fixed concentration, smaller particle sizes can be regarded as more stable. In other words, it is directly related to the effective scattering cross‐section. When the nanomaterial conditions of two samples are similar, the difference in correlation values remains small at short time lags, whereas at longer time lags, the relative stable versus unstable conditions of the two samples can be clearly discriminated (Figure 1d).

First, to confirm that the multiple light scattering occurring at the metal surface of the sample holder is beneficial for nanomaterial detection in liquids, we measured 200 nm polystyrene (PS) bead samples at a concentration of 10^8^ particles/mL and performed temporal correlation analysis. Figure 2a,d show a laser speckle image obtained using the metal sample holder and an image of the light transmitted directly through a glass vial, respectively. In the speckle image generated by multiple light scattering, the magnified view on the right demonstrates that a uniform speckle pattern is formed in each region. In contrast, in the absence of multiple light scattering, the image exhibits diffraction fringes due to the glass vial and streak‐like features caused by the flows of the PS beads. As can be seen in Videos S1 and S2, the laser speckle image exhibits continuous temporal variations, whereas in the nonmultiple‐scattering case, the overall diffraction pattern remains stable and only the features associated with PS bead motion change over time. Because the PS beads move very rapidly in the medium, their motion cannot be accurately resolved with an exposure time of 5 ms, leading to streak‐like patterns in the images.

**FIGURE 2:**
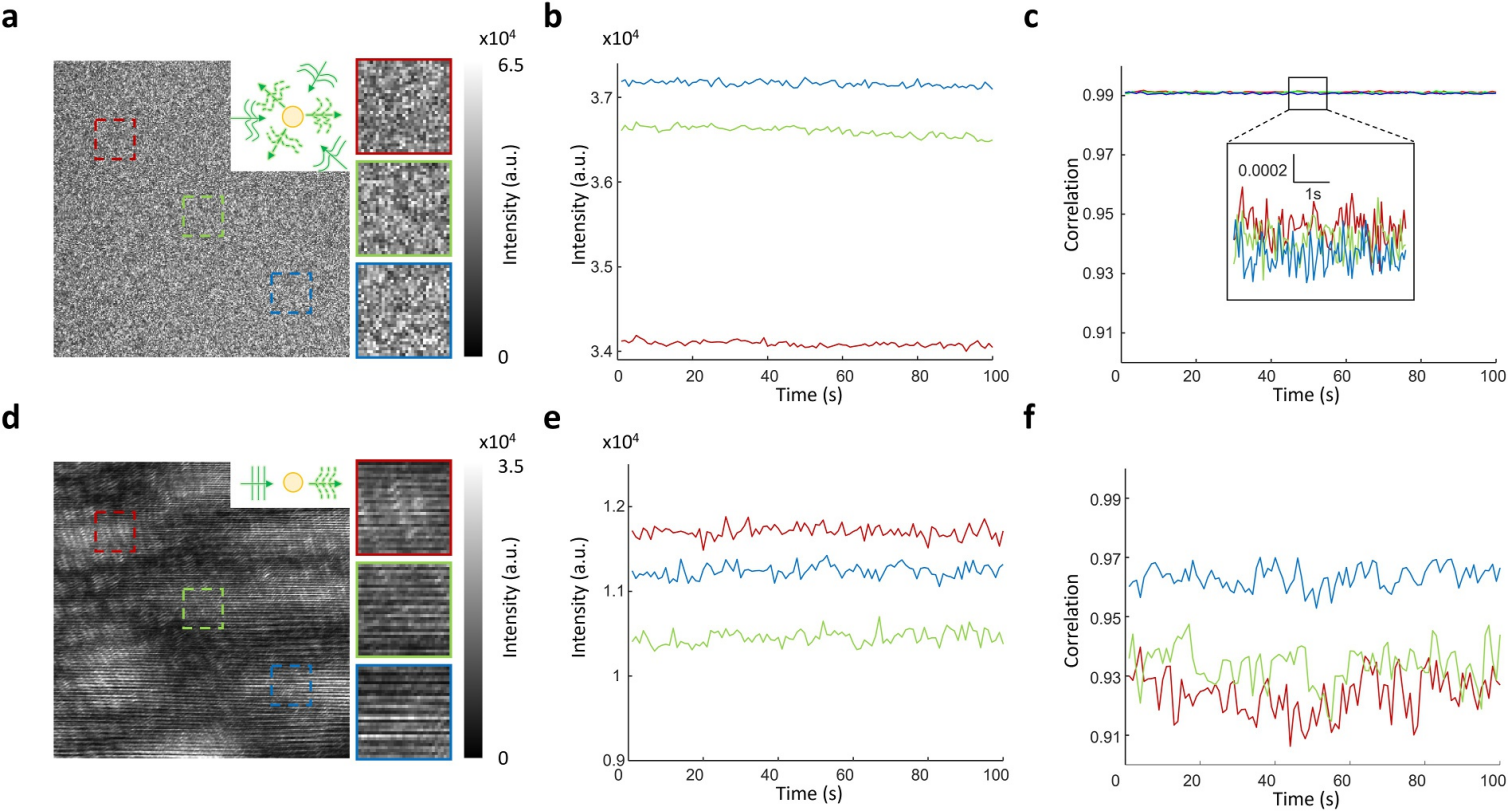
Investigation of the effects of multiple light scattering on the consistency of image analysis using 200 nm nanoparticles at a concentration of 10^8^ particles/mL. (a, d) Representative images obtained with (a) and without (d) harnessing multiple light scattering. The images on the right are magnified images of corresponding square regions indicated in the images on the left. (b, e) Average intensities over time corresponding the square regions shown in (a) and (d). (c, f) Temporal correlation analysis of three corresponding regions over time at a time lag of 5 ms.

To examine whether the speckle patterns yield stable analysis results regardless of the image region, we selected three regions of interest and evaluated the temporal changes of the average intensity (Figure 2b,e) and the temporal correlation at a time lag of 5 ms (Figure 2c,f). In both cases, the mean intensity differs from region to region, independent of the presence of multiple scattering. Although the brightness varies spatially, the speckle images exhibit relatively small temporal intensity fluctuations, whereas in the absence of multiple light scattering the intensity varies more dynamically over time. However, because the intensity distribution itself can change due to illumination conditions, we used correlation analysis to assess whether multiple light scattering provides an advantage for nanoparticle analysis in liquids. Using 2D Pearson's correlation, we quantified how the speckle pattern in each region changes over time. For the laser speckle images, the correlation analysis revealed nearly identical correlation values across regions, despite the differences in mean intensity. In the inset of Figure 2c, the variation in correlation among the regions is less than 0.0004, which indicates that the correlation analysis results are consistent. On the other hand, in the absence of multiple light scattering, the correlation values differ among regions, indicating that the streak patterns produced by PS beads floating in the liquid are not spatially uniform. These results demonstrate that laser speckle images generated by harnessing multiple light scattering provide stable and consistent analysis outcomes, independent of the specific image location.

We next examined whether the proposed method can be used to discriminate nanoparticle concentration. A stock solution of 200 nm PS beads at 10^10^ particles/mL was serially diluted to prepare samples with concentrations from 10^5^ to 10^8^ particles/mL, and phosphate‐buffered saline (PBS) without nanoparticles was used as a control. Figure 3 shows the results of 2D Pearson's correlation analysis of laser speckle images calculated at nine different time lags (5, 10, 15, 25, 50, 75, 100, 150, and 250 ms) for each concentration. At short time lags, all samples exhibit very high correlation values, indicating that the speckle patterns change only slightly within such short time intervals. In contrast, for time lags longer than 50 ms, the amount of decrease in correlation value becomes larger as nanoparticle concentration increases. In particular, at the longest time lag of 250 ms, samples with different nanoparticle concentrations from 10^5^ to 10^8^ particles/mL were clearly discriminated based on the decrease in correlation values. These results show that speckle imaging harnessing multiple light scattering and temporal correlation analysis can precisely discriminate the concentration of nanoparticles in liquids.

**FIGURE 3:**
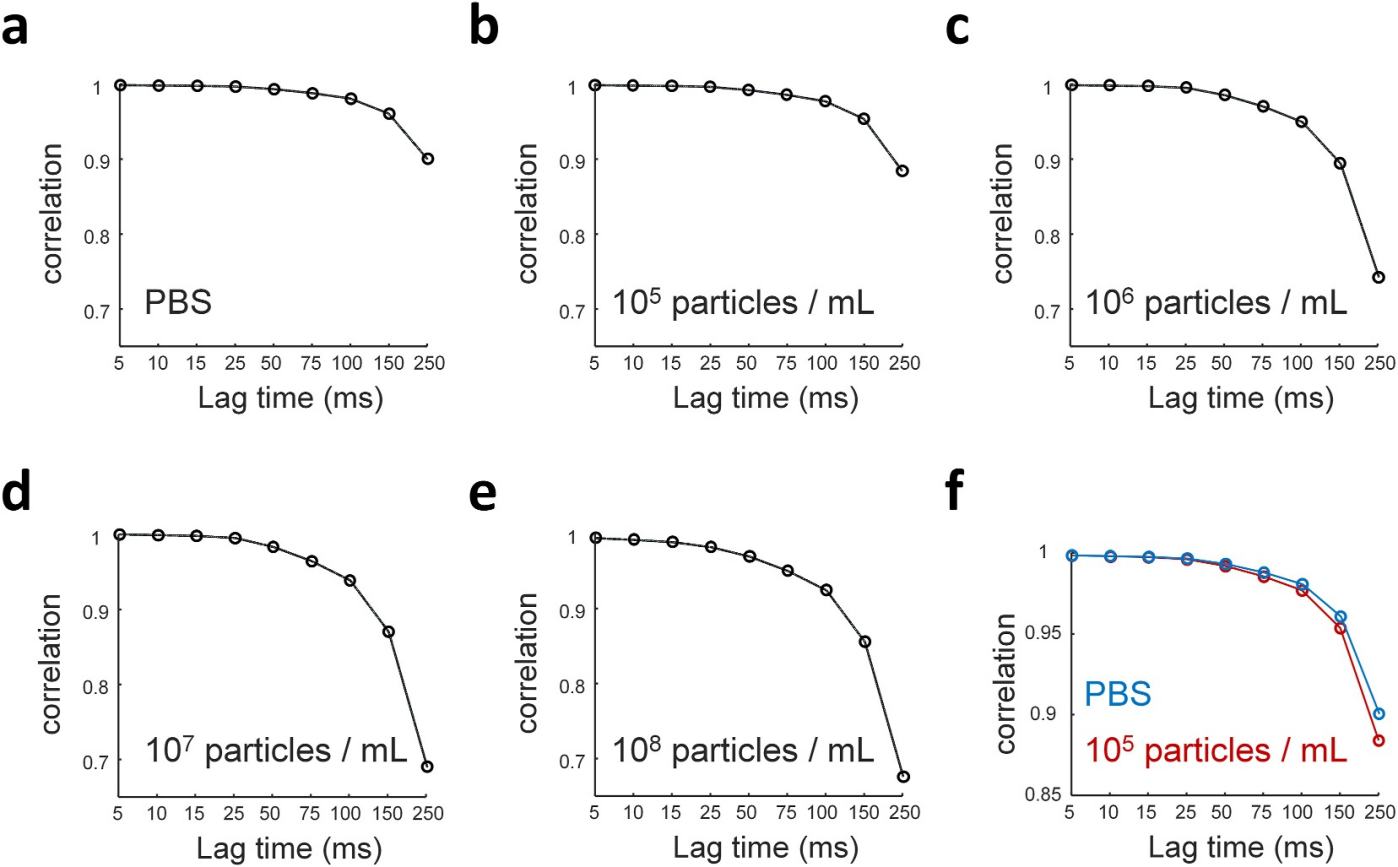
Nanoparticle concentration‐dependent decorrelation effects. (a–e) Correlation analysis of speckle images for 200 nm nanoparticles at different concentrations from 0 to 10^8^ particles/mL calculated at 9 different time lags (5, 10, 15, 25, 50, 75, 100, 150, and 250 ms). (f) Comparison of the correlation changes between PBS (no particles) and 10^5^ particles/mL.

Figure 3f compares the correlation analysis results for PBS (no nanoparticles) and the 10^5^ particles/mL sample. Up to a time lag of 50 ms, the correlation values are nearly indistinguishable; however, at longer time lags, a clear difference in correlation values emerges, demonstrating that the proposed method can reliably detect even low nanoparticle concentrations.

We then investigated how differences in nanoparticle size at a fixed concentration affect the speckle‐pattern analysis. Samples containing PS beads of 100, 150, 200, and 500 nm diameter were prepared at a concentration of 10^8^ particles/mL, and correlation analysis was performed at the eight time lags (5, 10, 15, 25, 50, 75, 100, and 150 ms). For the 100–200 nm PS bead samples, the decorrelation behavior with increasing time lag is similar among the three sizes and clearly distinguishable from that of PBS without nanoparticles. In contrast, for the 500 nm sample, the correlation value is already below 0.9 at the shortest time lag of 5 ms, indicating that the speckle pattern changes very rapidly. Figure 4f shows the correlation curves of the 100–200 nm PS bead samples on a scale from 0.85 to 1. For 100 and 150 nm beads, the correlation values are distinguishable, although correlation value differences are small until time lag of 25 ms. The 200 nm sample, however, shows correlation values that are clearly separated from those of the 100 and 150 nm samples. These results indicate that the temporal dynamics of the speckle pattern depends on nanoparticle size, which can be attributed to the increase in scattering cross‐section with particle size, leading to a huge influence on light scattering. In other words, the changes in scattering properties in the medium can be measured with high precision via the proposed multiple‐scattering–based speckle imaging method.

**FIGURE 4:**
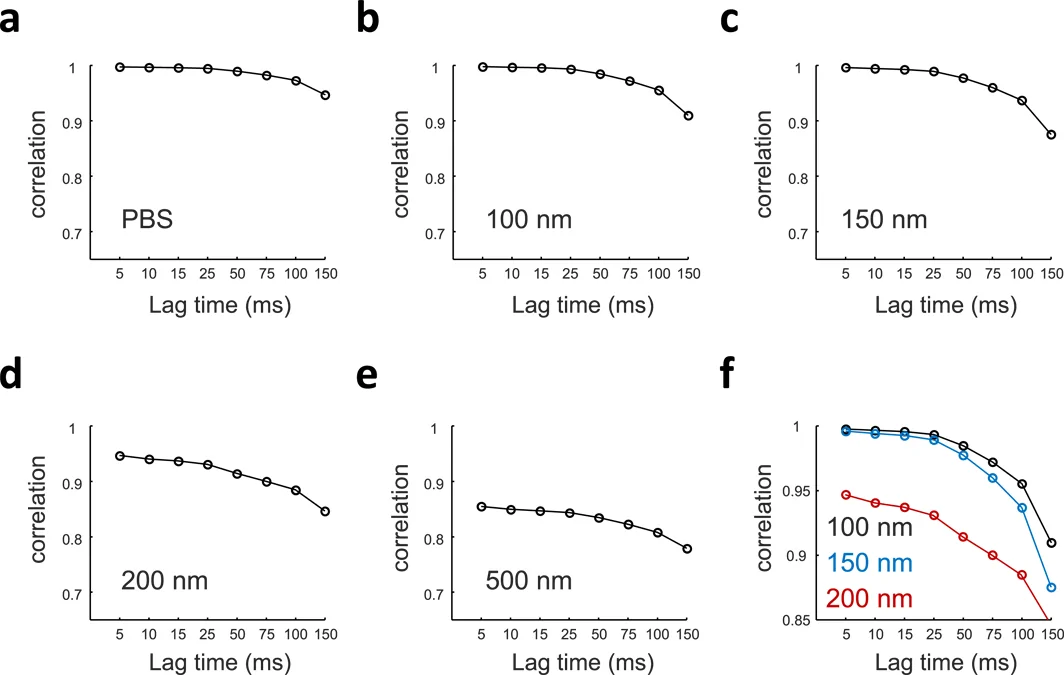
Nanoparticle size‐dependent decorrelation effects. (a–e) Correlation analysis of speckle images for nanoparticles with sizes from 100 to 500 nm with a fixed concentration of 10^8^ particles/mL calculated at 8 different time lags (5, 10, 15, 25, 50, 75, 100, and 150 ms). (f) Comparison of the correlation changes among three different nanoparticle sizes (100 nm, black; 150 nm, blue, and 200 nm, red).

This size‐dependent decorrelation behavior may appear inconsistent with Brownian motion theory, which predicts faster diffusion, and thus, faster intensity fluctuations for smaller particles. However, this apparent contradiction is resolved by considering the temporal resolution of the measurement. The proposed method acquires speckle images with an exposure time of 5 ms and a frame rate of 110 fps, which is orders of magnitude slower than the diffusion timescale of individual nanoparticles in solution. At this temporal resolution, the displacement of individual particles is averaged within each frame, and the frame‐to‐frame speckle decorrelation is no longer driven by single‐particle diffusion. Instead, it is dominated by the effective scattering cross‐section σ_scat: particles with larger σ_scat produce stronger perturbations to the multiply‐scattered light field per unit displacement, resulting in faster speckle decorrelation irrespective of their diffusion speed. FDTD simulations confirm this interpretation: as shown in Supporting Information S1: Figure S4a, σ_scat increases by several orders of magnitude from *d* = 100 nm to *d* = 500 nm at *λ* = 532 nm, and the corresponding electric‐field distributions (Supporting Information S1: Figures S4b–e) show substantially stronger scattered fields for larger particles. These simulation results are in quantitative agreement with the experimental observation that larger PS beads produce faster speckle decorrelation, establishing that σ_scat is the dominant determinant of speckle dynamics in the proposed measurement regime.

To provide a statistically rigorous empirical basis for size discrimination, we exploited the spatial uniformity of multiple‐scattering‐generated speckle fields to extract *N* = 100 independent correlation measurements from a single 500 × 500 pixel image by dividing it into 100 nonoverlapping 50 × 50 sub‐regions. As validated in Figure 2c, interregion variation in correlation values is less than 4 × 10^−4^. Thus, the temporal dynamics in each subregion are statistically independent, because multiple light scattering produces spatially homogeneous illumination with randomized phase. This spatial decomposition strategy thus converts a single measurement into a statistically sufficient dataset for calibration without additional acquisitions.

We define the control‐normalized decorrelation metric:

$$\Delta g_{2}\left(\tau =5ms\right)=1-\frac{g_{2,\text{sample}}\left(\tau =5ms\right)}{g_{2,\text{control}}\left(\tau =5ms\right)},$$

where *g*_2_, control is measured from particle‐free PBS under identical conditions. At *τ* = 5 ms, *Δg*_2_ is strictly monotone across all tested sizes (Supporting Information S1: Table S1).

The calibration reveals a regime‐dependent power–law relationship between *Δg*_2_ (*τ* = 5 ms) and particle diameter *d*:

$$g_{2}\left(\tau =5ms\right)=1.59\times 10^{-21}d^{8.41}$$

The size of an unknown sample can be estimated from a single *Δg*_2_ (*τ* = 5 ms) measurement that is consistent with ground‐truth values (Supporting Information S1: Figure S5). These results demonstrate that the proposed method achieves semiquantitative size determination in the 100–200 nm range under fixed‐concentration conditions, with prediction uncertainty of ± 7 nm at 100 nm and ± 2 nm at 150–200 nm.

For the technical evaluation of the proposed method, we measured the same PS bead samples used in Figures 3, 4 with a DLS instrument. Supporting Information S1: Figure S5 shows the particle size distributions obtained by DLS. We found that for 100, 150, and 200 nm PS beads, reliable DLS results were obtained only at concentrations of 10^9^ particles/mL and above; at lower concentrations, the measurements were unavailable. This is consistent with previous reports that the practical detection limit of DLS is on the order of 10^8^ particles/mL. In contrast, for the 500 nm sample, DLS measurements were feasible starting from 10^8^ particles/mL. The larger scattering cross‐section of the 500 nm PS beads might enable the detection at lower concentrations using DLS. Furthermore, the size distributions of the 100 and 150 nm samples measured by DLS overlap significantly, which might be related to the reasons that the proposed method obtained similar correlation curves for these two sizes.

From these results, we found that the proposed method, just consisting of a laser, a CMOS camera, and a metal‐coated holder, outperforms DLS for detecting nanoparticles with low concentrations. In addition, the present method exploits multiple light scattering to provide stable and consistent analysis results that are independent of the measurement location. Thus, this cost‐effective, simple, compact optical system offers many advantages for practical nanomaterial sensing in liquids.

The quantitative capability of the proposed method depends on the availability of prior sample information, and we distinguish two operating modes accordingly. When neither particle size nor concentration is known in advance, the method operates as a comparative tool: the control‐normalized decorrelation metric Δg_2_ is strictly monotone with both increasing concentration (at fixed size; Figure 3) and increasing particle size (at fixed concentration; Figure 4 and Supporting Information S1: Table S1), enabling reliable ranking of samples without additional assumptions. This comparative capability is itself practically valuable in applications such as process monitoring, quality control, and contamination screening, where relative comparisons between samples are the primary need.

When prior information about one variable is available, the method enables semiquantitative analysis of the other. In particular, under the fixed‐concentration condition (10^8^ particles/mL), the empirical calibration model established here achieves size determination in the 100–200 nm range with 95% prediction intervals within ± 7 nm (Supporting Information S1: Table S1), which is sufficient to distinguish the principal nanoparticle size classes of practical interest. This is in conceptual analogy with DLS, which also requires prior knowledge (temperature, viscosity, refractive index) to convert autocorrelation decay times to quantitative size values via the Stokes–Einstein equation.

The simultaneous, prior‐free extraction of both particle size and concentration from a single decorrelation curve remains beyond the scope of this work, as the speckle dynamics encode the combined effect of scattering cross‐section (size‐dependent) [30] and particle number density (concentration‐dependent) [31]. Decoupling these two contributions for samples of unknown composition, or for mixtures of multiple particle sizes will require either sequential measurements under systematically varied conditions or machine‐learning‐based inverse analysis of the full multitime‐lag decorrelation curve. We are actively pursuing this extension as follow‐up research.

## 3 Conclusion

The proposed method enables precise detection of nanomaterials in liquids by analyzing speckle patterns acquired with a simple optical setup. Under fixed‐concentration conditions, the empirical calibration model achieves semiquantitative size determination. Unlike DLS, the method does not require prior knowledge of temperature, viscosity, or optical constants, and can be implemented with low‐cost, simple optics without high‐speed imaging.

In applications where nanomaterial detection and comparison between samples are needed under fixed environmental conditions, this method can be highly useful. Because each speckle image represents a superposition of many scattered light fields, it inherently encodes information related to both nanoparticle size and concentration; however, temporal correlation analysis alone cannot fully decompose this information, as individual speckle fluctuations driven by nanoparticle dynamics are mixed with background noise and instrument‐induced variations that are difficult to separate analytically. AI‐based analysis could overcome this limitation such as deep learning models trained to distinguish genuine speckle dynamics from noise contributions across the full spatiotemporal speckle dataset, enabling simultaneous, prior‐free extraction of both particle size and concentration from a single measurement. This represents a promising direction for extending the proposed platform toward fully quantitative nanomaterial characterization.

## 4 Experimental Section

### 4.1 Optical Setup

The optical setup consisted of a laser source, sample holder, and a detector. The sample holder was a custom‐machined aluminum holder incorporating an internal spring–plunger mechanism to prevent lateral movement and maintain stable positioning during measurement. The sterile glass vial (SVC‐C1236, Hubena Inc.) with 2.3 mL volume was loaded in the sample holder. A diode‐pumped solid‐state (DPSS) laser (Cobolt Samba, Cobolt AB) emitting at 532 nm directly illuminated to the sample holder, and a CMOS camera (Blackfly S BFS‐U3‐04S2M, FLIR Inc.) was placed at the output port of the sample holder to capture speckle images. Light enters through the input window on the side of the holder and exits through the output window on the opposite side wall; the CMOS camera is placed laterally facing this side aperture, not the top of the vial.

### 4.2 Image Acquisition Process

Samples were prepared in glass vials and thoroughly agitated prior to each measurement to ensure uniform nanoparticle dispersion. The vial was then positioned within the sample holder, and the image acquisition was performed using MATLAB. Each measurement was recorded for a duration ranging from 1 to several minutes. To maintain signal integrity and minimize external noise, the entire experimental setup was housed in a controlled environment shielded from ambient light, airflow, and mechanical vibrations using a pneumatic vibration‐isolation table.

### 4.3 Nanoparticle Sample Preparation

Polystyrene counting standard particles (CD Bioparticles) were used as nanoparticle samples. Samples prepared via serial 1:10 dilutions using a dispersion medium (PBS with 0.01% Tween 20) identical to storage buffer. For each measurement, 2.5 mL of the diluted sample was freshly prepared in a glass vial and experiments were immediately performed.

### 4.4 DLS Measurement

Hydrodynamic diameters were measured using a Zetasizer Nano S (Malvern Panalytical) equipped with a 4 mW He–Ne laser (λ=633nm). Measurements were performed at a fixed backscattering angle of 173° to minimize multiple scattering effects. The measurement protocol consisted of three independent sets, with each set comprising 10 individual runs to ensure statistical significance. The Z‐average diameter and polydispersity index (PDI) were derived from the autocorrelation function via the Cumulants analysis method using the Zetasizer software. The refractive index and viscosity of the PBS‐based dispersion medium were set to 1.33 and 0.8872cP, respectively.

### 4.5 Temporal Correlation Analysis of Speckle Patterns

Temporal fluctuations in speckle patterns due to motion of nanoparticles were quantified by calculating frame‐to‐frame correlation coefficients using the two‐dimensional Pearson correlation coefficient. The correlation between successive frames $I_{t}$ and $I_{t+1}$ was computed by MATLAB (corr2 function, MathWorks, USA) as follows:

$$g_{2}\left(t\right)=\frac{\Sigma_{x,y}\left[I_{t}\left(x,y\right)-{\bar{I}}_{t}\right]\left[I_{t+1}\left(x,y\right)-{\bar{I}}_{t+1}\right]}{\sqrt{\Sigma_{x,y}{\left[I_{t}\left(x,y\right)-{\bar{I}}_{t}\right]}^{2}}\sqrt{\Sigma_{x,y}{\left[I_{t+1}\left(x,y\right)-{\bar{I}}_{t+1}\right]}^{2}}}$$

where ${\bar{I}}_{t}$ and ${\bar{I}}_{t+1}$ are the average pixel intensities. This method was chosen due to its established efficacy in characterizing subtle particle dynamics in speckle analysis. A total of N=6600 frames (∼60 s at 110 fps) were analyzed, producing a correlation time series defined by $t_{i}=i/f_{\text{cam}}$ ($f_{\text{cam}}$ = 110 fps).

## Author Contributions

J.Y. designed the study. J.K., Y.K., J.W.K., and J.Y. performed the acquisition of experimental data. H.N.C. and I.H.L. performed D.L.S. experiments. Y.K. performed numerical analysis of OPL enhancement. J.H., S.Y., C.Y.Y. and J.Y. analyzed the data. All authors wrote the manuscript.

## Funding

This research was supported by Global—Learning & Academic research institution for Master's·PhD students, and Postdocs (G‐LAMP) Program of the National Research Foundation of Korea (NRF) grant funded by the Ministry of Education (No. RS‐2023‐00285390).

## Conflicts of Interest

Kyoungman Cho and Jonghee Yoon have financial interests in The Wave Talk Inc., a company that commercializes laser speckle imaging instruments. The authors declare no conflicts of interest.

## Supporting information


Supporting Information S1

**Video S1:** Timelapse speckle images obtained using the metal‐coated sample holder with 200 nm polystyrene bead suspension at a concentration of 10⁸ particles/mL. Continuous temporal variations in the speckle pattern are visible throughout the recording, reflecting the multiple‐scattering‐mediated sensitivity of the system to nanoparticle motion.

**Video S2:** Timelapse images obtained without multiple light scattering (direct transmission through a glass vial containing 200 nm polystyrene beads at 10⁸ particles/mL). The overall diffraction pattern remains largely stable, with only localized streak‐like features associated with individual PS bead motion visible over time, in contrast to the global speckle dynamics observed in Video S1.

## Data Availability

The datasets and code used in this work are not publicly available due to further ongoing research. However, access may be granted to researchers upon reasonable request.
