# Supplementary material for: Development of a Lensless Compact Optical System for Ultrasensitive Nanomaterial Detection in Liquid Media via Exploiting Multiple Light Scattering
Source: Nanophotonics. 2026 Jul 5;15(13):e70193. doi: 10.1002/nap2.70193 (PMC13642118; doi:10.1002/nap2.70193)
Supplement: Supplementary file 1 [file NAP2-15-e70193-s001.docx]

Supplementary Information

**Development of a lens-less compact optical system for ultrasensitive nanomaterial detection in liquid media via exploiting multiple-light scattering**

Jeonggyo Kim^1,+^, Youngsoo Kim^2,+^, Jun-Won Kook^2^, Hae-Nam Choi^1^, Kyoungman Cho^3^, In-Hwan Lee^4^, Jongkook Hwang^1.5^, Sungju Yu^1.4^, Chung-Yul Yoo^1,4^, and Jonghee Yoon^1,6,*^

^1^Department of Energy Systems Research, Ajou University, Suwon, 16499, South Korea

^2^Ajou Energy Science Research Center, Ajou University, Suwon, 16499, South Korea

^3^The Wave Talk., Inc., Jinri Hall, 193, Munji-Ro, Yueseong-Gu, Daejeon, 34051, Republic of Korea

^4^Department of Chemistry, Ajou University, Suwon, 16499, South Korea

^5^Department of Chemical Engineering, Ajou University, Suwon, 16499, South Korea

^6^Department of Physics, Ajou University, Suwon, 16499, South Korea

[jyoon48@ajou.ac.kr](mailto:jyoon48@ajou.ac.kr)

**S1. Optical setup and representative speckle images acquired at different camera positions**

Figure S1. (a) (a) Schematic of the lens-free optical setup, consisting of a 532 nm DPSS laser, two mirrors, a custom aluminum sample holder, and a CMOS camera placed directly facing the side output aperture without any imaging optics. (b) Representative speckle images acquired at camera-to-sample distances of 0, 1, 2, and 5 cm. Speckle grain size increases proportionally with distance, confirming that well-developed speckle patterns form without any lens or focusing element.

**S2. Monte Carlo simulation of light distribution in cavities with mirror-like and diffusive surfaces**

**Monte Carlo Ray-Tracing Simulation**

A simple two-dimensional Monte Carlo ray-tracing simulation was performed to examine the light distribution inside the sample holder cavity. The cavity was represented as a rectangular cross-section (width $W$= 13 mm, height $H$= 25 mm) with an input port (width 1mm) on the lower-left wall and an output port (width 1 mm) on the upper-right wall. A total of $N$= 10⁵ rays were launched from uniformly distributed positions along the input port. The initial propagation direction of each ray was set to the +$x$ direction with an angular spread of ±$\theta_{div}$ sampled from a uniform distribution, where $\theta_{div}$was fixed as 0.1°.

At each wall intersection, the ray energy was multiplied by the reflectance $R_{Al}$= 0.99. The reflection direction was determined by stochastic branching based on the total integrated scatter (TIS), calculated from the Bennett–Porteus formula:

$$TIS=1-exp\left[ -\left( \frac{4\pi R_{q}}{\lambda} \right)^{2} \right]$$

where $R_{q}$is the root-mean-square surface roughness and $\lambda$= 532 nm is the laser wavelength. With probability TIS, the ray was reflected in a random direction sampled from a two-dimensional Lambertian distribution (cosine-weighted about the surface normal); with probability 1 − TIS, specular reflection was applied. Simulated conditions comparable to CNC-machined aluminum, $R_{q}$was set to 1.8 μm, yielding TIS ≈ 1.0. All reflections were Lambertian. For comparison, an ideal mirror surface ($R_{q}$ = 0, TIS = 0) was also simulated, in which all reflections were specular. Each ray was traced until it escaped through a port or its energy fell below 10⁻⁴ of the initial value. The irradiance distribution was recorded on a grid of 650 × 1250 cells (10 μm resolution) by accumulating the weighted path contributions along each ray segment.

Figure S2. Monte Carlo ray-tracing simulation of photon path distributions inside the sample holder cavity under two limiting surface conditions. Top row: specular (mirror-like) surface (R_q = 0), in which photons propagate along a narrow geometric path and the illuminated volume remains confined near the input port even after many reflections. Bottom row: diffuse surface (R_q = 1.8 μm, TIS ≈ 1.0), representative of the unpolished aluminum holder, in which photons redistribute progressively throughout the entire cavity cross-section with increasing number of reflections. Columns show cumulative photon distributions after the initial pass, 1st reflection, ≤10 reflections, and all reflections (left to right). The color scale indicates normalized irradiance. Green and cyan markers indicate the output and input ports, respectively. The diffuse surface fills the entire cavity volume, providing the spatially uniform illumination that underlies the sensitivity enhancement of the proposed method.

**S3. Finite-Difference Time-Domain (FDTD) simulation of two-dimensional cavity to provide mechanistic insights into cavity-induced reflection/multiple-scattering effects.**

Figure S3 illustrates the two-dimensional electromagnetic simulations conducted to investigate how the reflective cavity walls promote multiple scattering and recirculation of light in the proposed sample holder. Because full three-dimensional modeling of the experimental geometry is computationally expensive and geometrically complex, we used a scaled-down, simplified two-dimensional cross-section of a circular aluminum cavity filled with water and containing multiple PS nanoparticles. This model was used to support the physical basis of the proposed structure by isolating the effects of cavity reflections and changes in particle size/number on the resulting field distributions.

The cavity walls were modeled as aluminum with frequency-dependent optical constants [S1]. To incorporate material dispersion in the time-domain simulations, the complex permittivity of Al was implemented using a Drude–critical points (Drude-CP) model fitted to the reference optical constants. The interior medium was water with a refractive index of *n* = 1.3337, and PS nanoparticles with a refractive index of *n* = 1.5983 were randomly distributed inside the cavity [S2, S3]. Simulations were performed for different particle numbers (*N* = 10, 20, and 30) and particle diameters (*d* = 100 and 500 nm) to examine how both parameters influenced the scattered field.

In the Drude–critical points (Drude-CP) dispersion model, the complex permittivity is expressed as

$$\varepsilon\left( \omega\right)=\varepsilon_{\infty}-\frac{\omega_{p}^{2}}{\omega^{2}+i\gamma\omega}+\sum_{k} A_{k}\Omega_{k} \left( \frac{e^{i\phi_{k}}}{\Omega_{k}-\omega-i\Gamma_{k}}+\frac{e^{-i\phi_{k}}}{\Omega_{k}+\omega+i\Gamma_{k}} \right),$$

where $\varepsilon_{\infty}$ is the high-frequency permittivity, $\omega_{p}$ and $\gamma$ are the plasma frequency and Drude damping constant, and $A_{k}$, $\Omega_{k}$, $\Gamma_{k}$, and $\phi_{k}$ represent the amplitude, resonance frequency, damping, and phase of the *k*-th critical-point term. The Drude-CP parameters were fitted to the reference optical constants for Al [S1] and then used in the FDTD simulations following standard Drude-CP implementations [S4,S5].

Excitation was provided by an electric dipole pulse placed near one end of the cavity, emitting a broadband temporal waveform centered at 532 nm with a full width at half maximum of 20 nm. After the excitation source was switched off, the time evolution of the electromagnetic field inside the cavity was monitored over the two-dimensional (2D) simulation domain, and the maximum electric-field amplitude at each 2D spatial point was recorded to generate the mode profiles shown in Figure S3(b–h).

The resulting field distributions demonstrate that increasing the particle size (*d*) or number (*N*) leads to more complex spatial interference patterns and enhanced field fluctuations in the cavity. This behavior arises from the accumulation of phase perturbations induced by repeated scattering events and multiple reflections from the cavity walls, which recirculate light and effectively amplify weak local scattering into cavity-wide interference changes. Importantly, these simulations show that the reflective cavity converts local particle-induced scattering events into global modifications of the electromagnetic field, thereby providing physical support for the proposed structure and its enhanced sensitivity to nanoparticle size and concentration.

Together, the results in Figure S3 provide a physical basis for understanding how the custom cavity geometry enhances sensitivity to nanoparticle size and concentration by promoting a multiple-scattering regime with strong field randomization and phase accumulation.

Figure S3. (a) Two-dimensional cavity simulation domain presented as a scaled-down and simplified model owing to the computational cost and geometrical complexity of the full experimental configuration. The simulations were intended to provide mechanistic insights into cavity-induced reflection/multiple-scattering effects rather than to reproduce the exact experimental speckle statistics. The mode profiles of the scattered field were calculated for polystyrene (PS) nanoparticles of varying sizes and numbers embedded in a water-filled aluminum cavity with a diameter of 18 μm. (b) Two-dimensional (2D) spatial distribution (2D map) of the maximum electric-field amplitude measured after excitation by a dipole pulse applied at one end of the cavity and after the source was switched off. (c–d) Maximum electric-field amplitude mode profiles (2D maps) for N = 10 with particle diameters of d = 100 nm and 500 nm, respectively. The corresponding results for N = 20 and N = 30 are shown in (e–f) and (g–h), respectively.

**S4. FDTD simulation of variation in the scattering cross-section of PS nanoparticles as a function of particle diameter and wavelength**

Figure S4 presents the FDTD simulations performed to clarify the particle-size-dependent scattering behavior of PS nanoparticles embedded in water [S6, S7]. Simulations were performed using an in-house (homemade) FDTD implementation to calculate both the scattering cross-section spectra and the spatial distributions of the scattered electric field, which has been used and validated in our previous light-scattering studies [S8-S10]. In the simulations, spherical PS nanoparticles with diameters ranging from 100 to 500 nm were placed in a homogeneous aqueous medium. The refractive indices were held constant as *n*_PS_ = 1.5983 for polystyrene [S2] and *n*_water_ = 1.3337 [S3] (values at 532 nm), because their dispersion is small over the wavelength range considered here. The incident field was modeled as a linearly polarized plane wave propagating along the *z*-axis, with an electric field polarized along the *x*-axis. Perfectly matched layer (PML) boundary conditions were applied in all directions to avoid artificial reflections from the simulation boundaries.

The scattering cross-section shown in Figure S4(a) was calculated using the total-field scattered-field (TFSF) method. A closed integration surface was placed in the scattered-field region to capture only the scattered power, which was obtained by integrating the outward Poynting vector over the surface and normalizing it to the incident intensity. The wavelength-dependent simulations revealed a strong increase in the scattering cross-section with increasing particle diameter, particularly in the visible regime at approximately 532 nm.

Figures S4(b–d) show representative spatial mode profiles of the scattered electric-field amplitude at a wavelength of 532 nm for particle diameters of *d* = 100, 150, 200, and 500 nm, respectively. The field distributions illustrated that larger nanoparticles induced significantly stronger local electric-field perturbations and more extended scattering patterns. These results provide direct physical insight into how increasing the particle size enhances the light–matter interaction strength and increases the scattering contribution to the detected interference field, establishing the scattering cross-section as a key parameter governing the speckle signals in the experiments.

Figure S4. (a) Variation in the scattering cross-section of PS nanoparticles as a function of particle diameter and wavelength, calculated using FDTD simulations. Simulations were performed for PS nanoparticles (n_PS_ = 1.5983) embedded in water (n_water_ = 1.3337). (b–d) Spatial mode profiles showing the electric-field amplitude scattered by PS nanoparticles in water at a wavelength of 532 nm, corresponding to particle diameters of d = 100, 150, 200, and 500 nm.

S5. PS beads size distribution measured by DLS.

Figure S5. Hydrodynamic size distributions of polystyrene (PS) bead samples measured by DLS. Size distributions are shown for nominal diameters of 100, 150, 200, and 500 nm, all measured at a concentration of 10⁹ particles/mL — the minimum concentration at which reliable DLS results were obtainable for sub-500 nm particles. All measured Z-average diameters are in close agreement with the nominal values specified by the manufacturer, confirming that the samples are monodisperse and well-characterized. These DLS measurements serve as independent verification of the particle sizes used throughout this study.

Supplementary Videos.

Video S1. Time-lapse speckle images obtained using the metal-coated sample holder with 200 nm polystyrene bead suspension at a concentration of 10⁸ particles/mL. Continuous temporal variations in the speckle pattern are visible throughout the recording, reflecting the multiple-scattering-mediated sensitivity of the system to nanoparticle motion.

Video S2. Time-lapse images obtained without multiple light scattering (direct transmission through a glass vial containing 200 nm polystyrene beads at 10⁸ particles/mL). The overall diffraction pattern remains largely stable, with only localized streak-like features associated with individual PS bead motion visible over time, in contrast to the global speckle dynamics observed in Video S1.

**Table S1. Evaluation of the empirical model to estimate nanoparticle sizes from decorrelation curves.**

**Supplementary References.**

S1.	Lide, David R. 2006. “CRC Handbook of Chemistry and Physics”, 87th Edition, CRC Press.

S2.	Zhang, X., J. Qiu, X. Li, J. Zhao, and L. Liu. 2020. “Complex Refractive Indices Measurements of Polymers in Visible and Near-Infrared Bands.” Applied Optics 59, 2337–2344.

S3.	Hale, G. M., and M. R. Querry. 1973. “Optical Constants of Water in the 200-nm to 200-µm Wavelength Region.” Applied Optics 12, 555–563.

S4.	Chun, K., Kim, H., Kim, H., and Chung, Y. 2012. “PLRC and ADE Implementations of Drude–Critical Point Dispersive Model for the FDTD Method.” Progress In Electromagnetics Research 135, 373–388.

S5.	Shibayama, J., Watanabe, K., Ando, R., Yamauchi, J., and Nakano, H. 2012. “Frequency-Dependent Formulations of a Drude-Critical Points Model for Explicit and Implicit FDTD Methods Using the Trapezoidal RC Technique.” IEICE Transactions on Electronics E95-C(4), 725–732.

S6.	Taflove, Allen, Susan C. Hagness, and Melinda Piket-May. 2005. “Computational Electromagnetics: The Finite-Difference Time-Domain Method.” In The Electrical Engineering Handbook, 3:629–670.

S7.	Taflove, Allen, Ardavan Oskooi, and Steven G. Johnson. 2013. Advances in FDTD Computational Electrodynamics: Photonics and Nanotechnology. Artech House.

S8.	Hong, S., Lee, Y. J., Moon, K., Kim, Y., Shin, E., and Kwon, S.-H. 2019. “Far-Field Analysis on Reflecting Colors of Dielectric Nanosphere Metasurface.” Journal of Nanomaterials 2019, 6532967.

S9.	Hong, S., Kim, Y., Hong, S. H., Kim, B., and Kwon, S.-H. 2024. “Ultrahigh Quality Factor Cavity Based on Double Dielectric Nanocylinder Metasurfaces.” Chinese Journal of Physics 92 (December 2024), 1133–1143.

S10.	Hong, S. H., Hong, S., Kim, Y., Kim, B., and Kwon, S.-H. 2025. “Single-Layered All-Dielectric Chiral Metasurface via Mie Resonances.” Physical Review A 112, 043547 (October 2025).
